# Effects of Block Copolymer Terminal Groups on Toughening Epoxy-Based Composites: Microstructures and Toughening Mechanisms

**DOI:** 10.3390/mi14112112

**Published:** 2023-11-17

**Authors:** Gang Li, Wenjie Wu, Xuecheng Yu, Ruoyu Zhang, Rong Sun, Liqiang Cao, Pengli Zhu

**Affiliations:** 1 System Packaging and Integration Research Center, Institute of Microelectronics of Chinese Academy of Sciences, Beijing 100029, China; 2 Shenzhen Institute of Advanced Electronic Materials, Shenzhen Institutes of Advanced Technology, Chinese Academy of Sciences, Shenzhen 518055, China; wenj1998@mail.ustc.edu.cn (W.W.); xc.yu@siat.ac.cn (X.Y.);; 3 University of Chinese Academy of Sciences, Beijing 100049, China

**Keywords:** epoxy, block copolymer, induced microphase separation, fracture toughness, toughening mechanisms, digital image correlation

## Abstract

Despite the considerable research attention paid to block copolymer (BCP)-toughened epoxy resins, the effects of their terminal groups on their phase structure are not thoroughly understood. This study fills this gap by closely examining the effects of amino and carboxyl groups on the fracture toughness of epoxy resins at different temperatures. Through the combination of scanning electron microscopy and digital image correlation (DIC), it was found that the amino-terminated BCP was capable of forming a stress-distributing network in pure epoxy resin, resulting in better toughening effects at room temperature. In a 60 wt.% silica-filled epoxy composite system, the addition of a carboxyl-terminated BCP showed little toughening effect due to the weaker filler/matrix interface caused by the random dispersion of the microphase of BCPs and distributed silica. The fracture toughness of the epoxy system at high temperatures was not affected by the terminal groups, regardless of the addition of silica. Their dynamic mechanical properties and thermal expansion coefficients are also reported in this article.

## 1. Introduction

Epoxy resins and their composites are widely sought due to their excellent chemical resistance, novel mechanical and electrical insulation properties, and good adhesion to various materials. Moreover, the flexible processability of epoxy resin is not possessed by other thermosetting materials [1,2,3,4,5,6,7,8]. However, unmodified epoxy resin has relatively poor heat resistance and toughness. Thus, nano-silica-filled epoxy systems are created to overcome these disadvantages [9,10]. The addition of nano-silica can improve the storage modulus while significantly reducing the thermal expansion coefficient of epoxy materials so that they can be used as adhesives for electronic packaging [11].

Epoxy-resin-based composites are often used as underfill adhesives for electronic assembly for integrated circuit (IC) chips using advanced electronic packaging technology. They fill the gap between the chip and the substrate under capillary action. After curing, the thermal stress originally concentrated on the solder joints around the periphery of the chip can be redistributed to the chip, underfill material, substrate, and all solder joints. As semiconductor silicon technology develops towards nanometer feature sizes, 3D system-level packaging with large sizes, narrow pitch, and high interconnected I/O density have almost become an inevitable trend. The high heat caused by higher power consumption, the greater warping deformation caused by large-size chips, and the flow filling problems caused by a narrow pitch and high I/O density have posed more stringent challenges to the mechanical properties of epoxy composite underfill.

The toughening of epoxy systems has long been an activity of Interest [12,13,14,15,16], and the effects of various tougheners (e.g., reactive rubber [17,18,19,20], thermoplastic resin [21,22], and core–shell particles [23,24,25]) on their fracture toughness have been studied. However, the addition of these tougheners often decreases the storage modulus and glass transition temperature (Tg). Among the many toughening studies related to epoxy resin, research on nanodomain toughening agents in multi-BCPs has attracted the most attention [26,27,28,29,30,31,32,33,34,35,36]. Owing to their long-range ordered nanostructures, BCPs can generate dispersed nanodomains in epoxy resin, which can improve toughness while maintaining Tg and the storage modulus [37,38,39,40,41].

Poly(methyl methacrylate) (PMMA) and poly(n-butyl acrylate) (PnBA) comprise PMMA-b-PnBA-PMMA, an amphiphilic tri-BCP that is an important BCP toughener. Asada et al. studied the BCP-induced microphase separation process in epoxy in situ using small-angle X-ray scattering. They found that the separation of the microphase occurred in two steps. First, a micelle structure is formed through the self-assembly of the BCP in uncured epoxy resin. Next, the micelle grows during curing and forms miscible PMMA blocks embedded in the epoxy network [42]. Klingle et al. studied the effects of nanophase BCPs on fatigue crack propagation in alicyclic amine-cured epoxy, finding that the anti-fatigue crack propagation ability of nanophase BCPs is related to the shielding mechanism around the crack-tip area [43]. Kishi et al. studied the phase size and morphology of BCPs when using different curing agents, demonstrating that the miscibility between the PMMA segment of a tri-BCP and the epoxy resin/curing agent is the key factor in producing a nanophase structure in the blend, and the content and molecular weight of the PMMA-b-PnBA-b-PMMA tri-BCP can affect the morphology. It was found that the epoxy/tri-BCP alloy with a nanocylindrical phase has the highest fracture toughness [44]. By treating the polystyrene (PS)-based PS-b-PnBA-b-PS tri-BCP with hydrogen peroxide, George et al. obtained butadiene blocks with different degrees of epoxidation, which leads to macroscale–nanoscale phase separation in the epoxy resin. Phase separation induced by the reaction of the polystyrene phase and the self-assembly of the polybutadiene produces a nanostructure that significantly improves fracture toughness and impact strength [45,46]. Taylor et al. studied a modified anhydride-cured diglycidyl ether (DGEBA) epoxy resin by regulating the content of three different types of PMMA-b-PnBA-b-PMMA tri-BCPs and found two types of phase structures in which the nanoscale micelle structure is the most favorable for toughening. When the amount of BCP increases, the phase structure changes to a bi-continuous phase structure, and the fracture toughness increases significantly. However, the tensile and compressive properties decrease slightly [47]. In general, the research on the toughening of epoxy resin and its composite system has been limited to room temperature settings and has focused on the influence of molecular weight and block copolymer content on its phase structure. The influence of block copolymer terminating groups on phase structure and toughening mechanisms remains poorly understood.

In this study, the effects of PMMA-b-PnBA-b-PMMA tri-BCP phase structures with amino and carboxyl terminating groups on the fracture toughness of a diamine-cured epoxy resin system were compared. Additionally, the morphology and distribution of the toughening agent phase in the silica-filled epoxy system were examined. The effects of different group-terminated BCPs on the strain field around the crack tip of a single-sided notch cantilever beam (SENB) specimen were observed using digital image correlation (DIC). The toughening mechanisms of the two BCPs were compared based on microscopic fracture surface analysis and strain field characterization, and the fracture toughness of the composite system was investigated at high temperatures. The storage modulus, glass transition temperature, and thermal expansion coefficient of the material were evaluated, providing valuable guidance for the application of BCPs in electronic packaging.

## 2. Materials and Methods

### 2.1. Material

**Figure 1:**
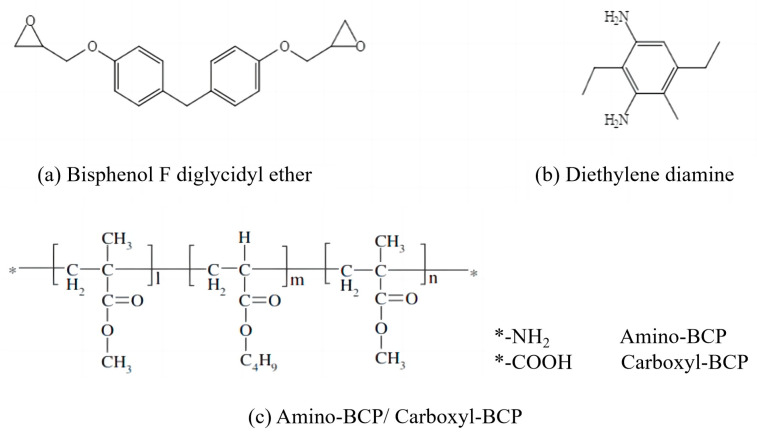
Chemical structures of (**a**) epoxy resin, (**b**) curing agent, and (**c**) BCPs.

The epoxy resin used in this study was a diglycidyl ether of bisphenol F precursor purchased from Hexion (Columbus, OH, USA) (epoxy equivalent: 142.857 g/eq), and diethylenediamine was purchased from Shanghai Aladdin Biochemical Technology Co., Ltd., Shanghai, China (DETDA, active amino hydrogen equivalent: 63 g/eq). Amino-terminated blocked tri-BCP (Amino-BCP) was purchased from Arkema (GRL, White Pigeons, France), and carboxyl-terminated tri-BCP (Carboxyl-BCP) was purchased from Kuraray Co., Ltd. (Tokyo, Japan). Spherical SiO_2_ with a particle size of approximately 900 nm (Suzhou Jinyi New Material Technology Co., Ltd., Suzhou, China) was used. The chemical structures of these materials are shown in Figure 1, and in Figure 1c, the asterisk sign represents the amino group of Amino-BCP and the carboxyl group of Carboxyl-BCP, respectively.

### 2.2. Preparation of Composites

**Figure 2:**
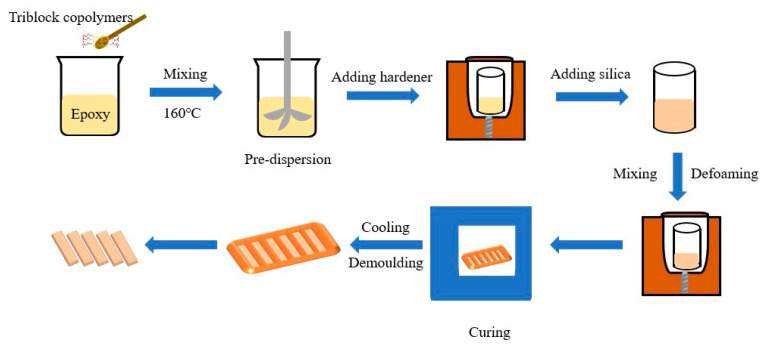
Schematic presentation of the processing and fabrication of composites.

First, the BCPs were dissolved in epoxy resin at 150 °C with a ratio of 5.714 g of BCP per 100 g of resin. The mixture was stirred for 30 min until clear, and the curing agent was added to the cooled premixture and mixed using a planetary mixer (FlackTek SpeedMixer, Landrum, SC, USA, DAC 600) at 1500 and 2000 rpm for 2 min. For the 60 wt.% silica-filled epoxy system, silica was added with a hardener, and the same mixing procedure was used to ensure that the filler was fully dispersed. After the equipment cooled, a 6 min vacuum defoaming process was performed at 800 rpm. The mixed liquid mixture was preheated on a hot stage at 60 °C for 3 min, followed by curing in a polytetrafluoroethylene mold at 165 °C for 2 h. After cooling for 30 min, the cured epoxy resin/composite specimens were removed from the mold. Figure 2 presents a flowchart of the sample preparation. For ease of reading, the relevant control groups are labeled and details are shown in Table 1.

### 2.3. Characterizations

The storage modulus and Tg of epoxy composites were tested using dynamic thermo-mechanical analysis (DMA, TA, New Castle, DE, USA, DMA850) in double-cantilever mode at 1 Hz and 3 °C/min. The sample size was 60 × 13 × 3 mm^3^, and the temperature range was 25–180 °C. The thermal expansion coefficients (CTEs) of the epoxy resin composites were determined using static thermomechanical analysis (TMA, TA, New Castle, DE, USA, TMA 450). A sample size of 5 × 5 × 4 mm^3^ and compression mode were adopted. The system was heated from 20 to 280 °C at a heating rate of 5 °C/min.

The fracture toughness was measured using an SENB test according to the ASTM E1820 standard [48] in a universal testing machine (SENS, Shenzhen, China, CMT4503). The critical stress intensity factors ($K_{IC}$) of the composites were computed using Equation (1):

(1) $$K_{IC}=\frac{P_{Q}}{{BW}^{\frac{1}{2}}}f(\frac{a}{W})$$

where *P_Q_* is the peak force required to propagate a crack, *B* is the specimen thickness, *W* is the specimen width, a is the crack length, and *f*(a/*W*) is the geometric factor. The sample was a rectangular parallelepiped with rounded edges measuring approximately 36 × 4 × 8 mm^3^. Prior to the test, a sharp pre-crack was initiated ahead of the machined notch in each specimen by tapping it with a fresh razor blade. The initial crack length, a, was measured using an optical microscope and satisfied the 0.45 ≤ *a*/*W* ≤ 0.55 length requirement. The loading rate was set at 0.5 mm/min.

The plane strain condition requires the specimen to satisfy the following conditions to ensure its validity:

(2) $$a\geq 2.5\left(\frac{K_{IC}}{\sigma_{y}}\right)\;$$

(3) $$Β\geq 2.5{\left(\frac{K_{IC}}{\sigma_{y}}\right)}^{2}$$

where $\sigma_{y}$ is the yield stress of the specimen, and $K_{IC}$ is its critical stress intensity factor.

The fracture surface of the cured epoxy resin composite was analyzed using a scanning electron microscope (SEM, Thermo Fisher Scientific, Waltham, MA, USA), and the phase structure of the BCP was observed. All samples were coated with a thin layer of sputtered gold to facilitate electrical conduction on the surface. The accelerating voltage was set at 10 kV.

In situ observation of the strain field at the tip of the spline crack during the fracture toughness test was performed using DIC (Yansuo Instrument Technology (Shanghai, China) Co., Ltd.) to obtain full-field displacement and strain distribution by comparing two images with different deformations [49,50,51].

## 3. Results

### 3.1. Dynamic Mechanical Property

**Figure 3:**
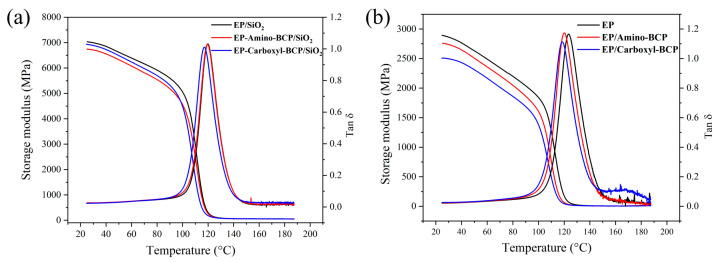
(**a**) Modulus and Tg of epoxy/BCPs; (**b**) modulus and Tg of epoxy/BCPs with 60 wt.% silica.

The storage modulus and Tg of both the epoxy resin/BCP and epoxy resin–silica/BCP systems were tested. As shown in Figure 3a, compared with EP, the storage modulus and Tg of EP/Carboxyl-BCP decreased by 11.9% and 5.11 °C, respectively. This may have been caused by the blocking effect of the dispersed phase on the crosslinking reaction. Notably, the storage modulus at room temperature and the Tg of EP/amino-BCP remained unchanged. The addition of 60 wt.% silica slightly changed the thermal mechanical properties of the epoxy/BCP composite system. As shown in Figure 3b, the storage modulus at room temperature and the Tg of the composites with different terminal groups of BCP were similar. The introduction of silica led to a decrease in Tg in both epoxy/BCP systems. This was mainly caused by the adverse effect of silica on the formation of epoxy resin crosslinking networks, which masked the influence of the introduced BCPs.

### 3.2. Coefficient of Thermal Expansion (CTE)

**Figure 4:**
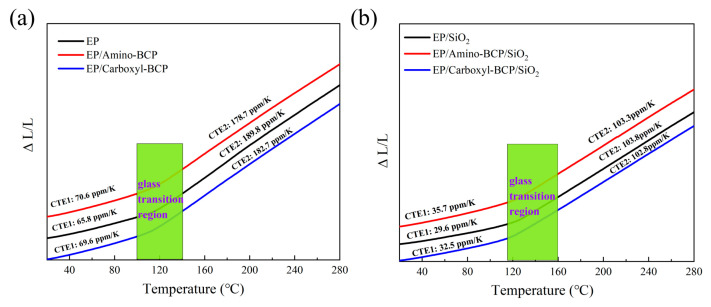
TMA results of epoxy/BCPs: (**a**) 0% silica system, (**b**) 60 wt.% silica system.

CTE is especially important for ensuring the suitability of epoxy composites in electronic packaging. Figure 4 shows the thermal expansion curves of the epoxy/BCP and epoxy resin–silica/BCP systems. The CTE1 and CTE2 values of each system are listed in Table 2.

Although the terminal groups were different, the addition of BCPs had a similar effect on the CTEs of the epoxy resins. The TMA results showed an increase in CTE1 and a decrease in CTE2 when both BCPs were introduced into the epoxy. The addition of the amino-terminated BCP led to a more significant increase of 4.8 ppm/K in CTE1 and a decrease of 11.1 ppm/K in CTE2, compared with neat epoxy. This is mainly caused by the lubricating effect of flexible BCPs in the epoxy resin matrix before Tg. However, after Tg, BCPs occupying a certain spatial position in the matrix can hinder the movement of chain segments in the matrix.

In the EP/SiO_2_ system, the addition of both types of BCPs had little effect on CTE2, as shown in Table 2. However, the improvement in the CTE1 of EP/amino-BCP/SiO_2_ was still significant, and was 3.2 ppm/K higher than that of EP/Carboxyl-BCP/SiO_2_. Therefore, it can be inferred that there were differences in the network structure formation of the epoxy resin between the two different terminal group BCPs, among which the structure of EP/amino-BCP/SiO_2_ was more conducive to the movement of chain segments.

### 3.3. Fracture Toughness

**Figure 5:**
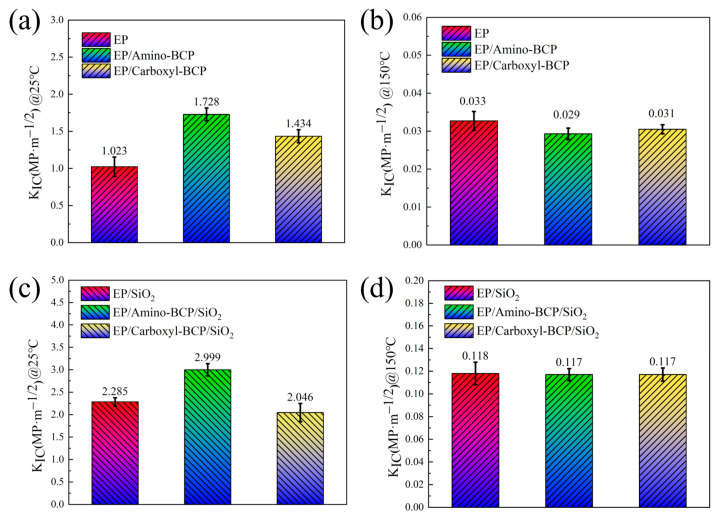
Fracture toughness of (**a**) epoxy/BCPs at 25 °C, (**b**) epoxy/BCPs at 150 °C, (**c**) epoxy/BCPs with 60 wt.% silica at 25 °C, and (**d**) epoxy/BCPs with 60 wt.% silica at 150 °C.

The fracture toughness results for the pure and silica-filled epoxy systems are shown in Figure 5. It can be seen that at room temperature, in the EP system, the addition of the amino- and carboxyl-terminated BCPs improved fracture toughness by 68.9 and 40.2%, respectively. In the EP/SiO_2_ system, the addition of the amino-terminated BCP caused an increase of 1.5 MPa·m^1/2^ in its fracture toughness, whereas the addition of the carboxyl-terminated BCP reduced the facture toughness by 0.5 MPa·m^1/2^. At 150 °C, the effect of the BCP on both pure and silica-filled epoxy systems disappeared. This may have been caused by the significant decrease in storage modulus after the epoxy system reached Tg (around 120 °C). Note that the fracture toughness of both the resin and composite systems at high temperatures was significantly lower than that at room temperature; therefore, the difference in the BCP systems with different terminal groups was no longer obvious, suggesting that there was a significant difference in the toughening mechanism at room and high temperatures. This can be explained by the SEM observations of the fracture surfaces of the samples at different test temperatures, which are analyzed in detail in Section 3.4.

### 3.4. Fracture Surface Analysis 

**Figure 6:**
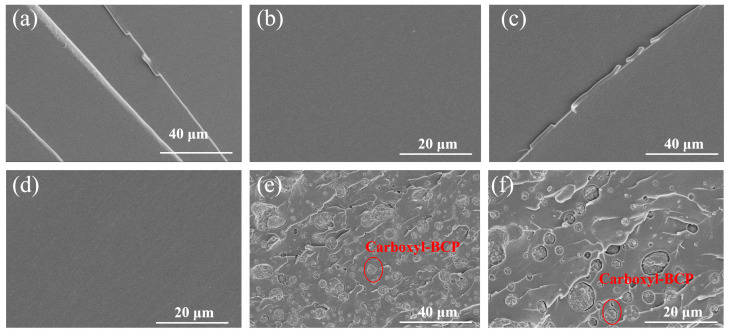
Fracture surface of the SENB specimens at 25 °C: (**a**,**b**) fracture surface of epoxy; (**c**,**d**) fracture surface of EP/Amino-BCP; (**e**,**f**) fracture surface of EP/Carboxyl-BCP.

**Figure 7:**
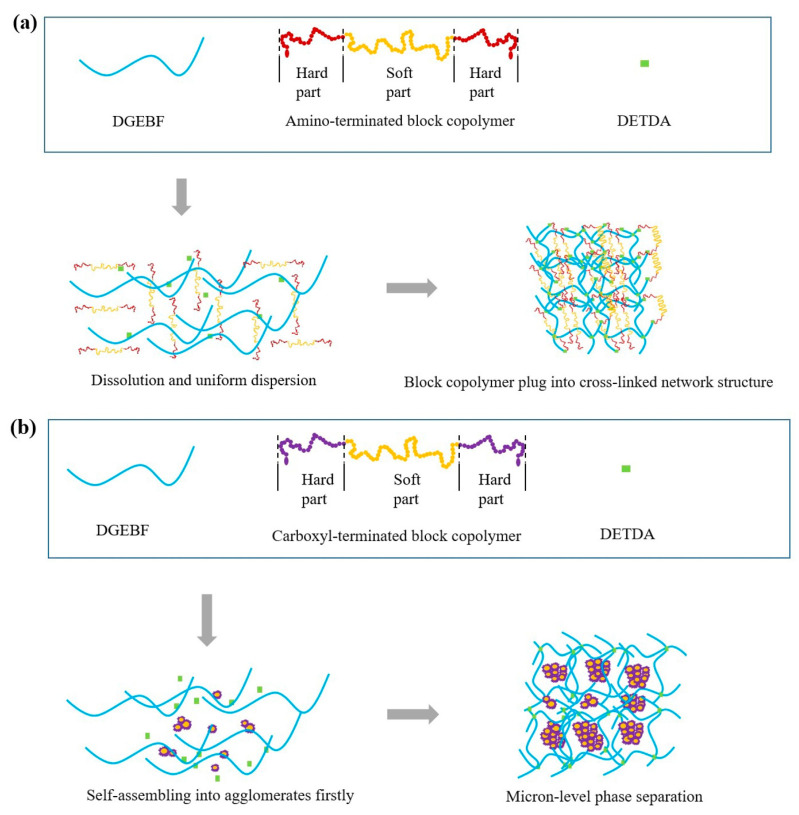
Curing reaction and material structure description: (**a**) epoxy resin/amino-terminated BCP system and (**b**) epoxy resin/carboxyl-terminated BCP system.

The fracture surfaces of the broken room-temperature SENB specimens were observed using SEM. As shown in Figure 6, in the EP system, pure epoxy resin exhibits a brittle fracture. It can be seen from the cross-section that the crack is composed of a main crack and some small branch cracks, and its fracture surface is relatively smooth. The addition of the amino-terminated BCP had little effect on the fracture surface. The fracture surface of the amino-terminated BCP system was generally as smooth as that of pure epoxy, and no obvious microphase separation was observed. However, the fracture surface of the EP/carboxyl-BCP was rough, and a micron-phase structure was observed. Through fracture surface analysis of the SENB specimens at room temperature, it was observed that EP/amino-BCP and EP/carboxyl-BCP formed different structures in the epoxy resin. This is closely related to the differences in the terminal groups of the BCP. In EP/amino-BCP, the terminal groups can be integrated into the molecular chains of the epoxy resin. As shown in Figure 7, a large number of flexible chain segments were connected to the original crosslinked network, which restricted the phase separation of EP/amino-BCP. This structure was more effective for stress transmission in the matrix and improved fracture toughness. For EP/carboxyl-BCP, owing to the presence of the amine curing agent, the carboxyl-terminated BCP underwent self-assembly, followed by agglomeration in the early stage of the reaction, resulting in significant phase separation in the epoxy resin matrix [31,36,46].

**Figure 8:**
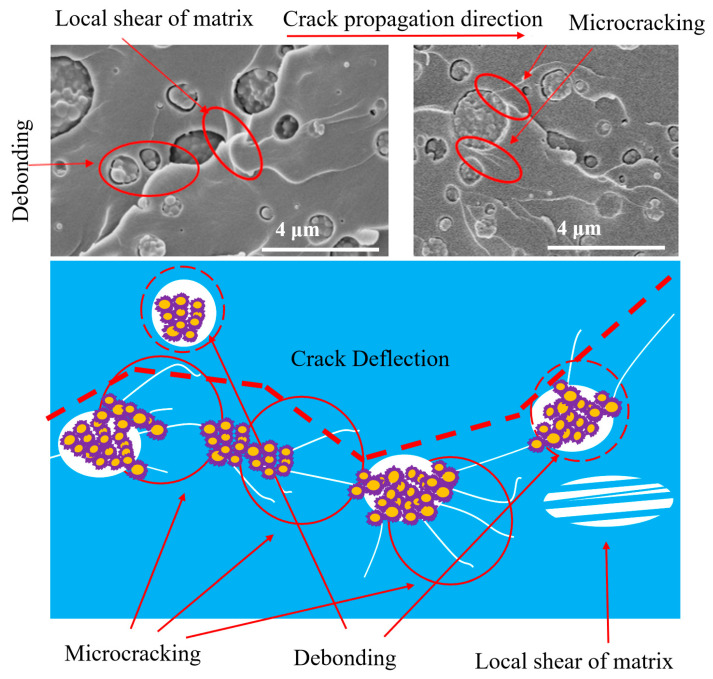
Proposed mechanism for explaining the increase in fracture toughness of the epoxy resin/carboxyl-terminated BCP system.

Further analysis of the fracture surface of the carboxyl-terminated BCP system showed that after the crosslinking and curing reactions, a significant number of island-phase structures of different sizes were distributed in the epoxy resin matrix. These dispersed phases act as fillers. As shown in Figure 8, during the fracture process, the dispersed phase can induce shear deformation of the matrix. Additionally, microcracking and crack deflection caused by the island phase can improve fracture toughness [34,36,43].

**Figure 9:**
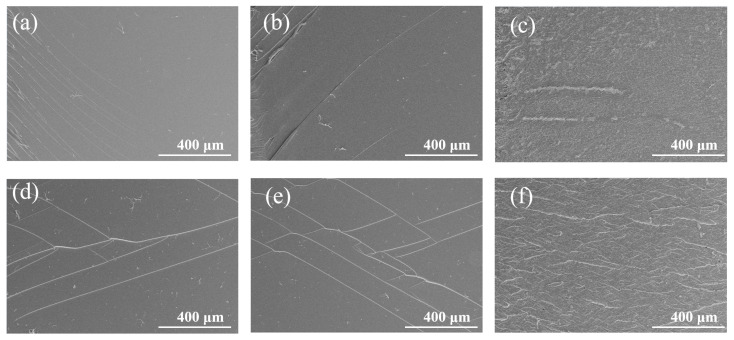
Fracture surface of the SENB specimens tested at 25 and 150 °C: (**a**) epoxy, (**b**) EP/Amino-BCP, and (**c**) EP/Carboxyl-BCP were tested at 25 °C; (**d**) epoxy, (**e**) EP/Amino-BCP, and (**f**) EP/Carboxyl-BCP were tested at 150 °C.

A comparison of the fracture surface results (Figure 9) at room and high temperatures showed that the main difference was that the matrix exhibited more bulk deformation during high-temperature testing at low magnification. Amino-BCP is connected to the crosslinked network of epoxy resin, and a flexible segment structure is embedded in the original crosslinked network structure. This structure has a forced inclusion effect and can stabilize the combination of different components. In addition, amino-BCP has good compatibility with epoxy resin, constraining phase separation and effectively constraining crack expansion. The main toughening method comes from the shear deformation effect of this structure on the matrix. The carboxyl-terminated triblock copolymer C is affected by the amine curing agent in the homogeneous mixture, and agglomeration occurs first during the crosslinking and curing process, resulting in the appearance of “island phase” structures of different sizes. These dispersed phases act as stress concentration, and debonding and microcracks can occur during the fracture process, hindering crack propagation. This is related to the chain segment movement above Tg and the matrix undergoing high elastic deformation. Although the matrix deformation of the epoxy resin changed significantly at high temperatures, the significant reduction in the storage modulus resulted in a significant decrease in the fracture energy absorption capacity of the matrix. Therefore, BCPs with different terminal groups at high temperatures led to a limited improvement in the fracture toughness of the epoxy resin.

**Figure 10:**
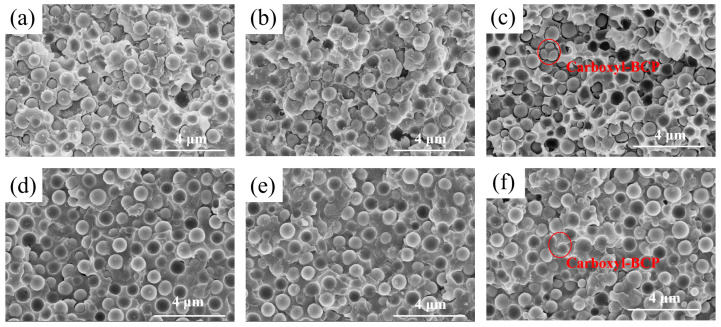
(**a**) Fracture surface of EP/SiO_2_ at 25 °C, (**b**) fracture surface of EP/Amino-BCP/SiO_2_ at 25 °C, (**c**) fracture surface of epoxy/carboxyl-terminated BCP with 60 wt.% SiO_2_ at 25 °C, (**d**) fracture surface of EP/SiO_2_ at 150 °C, (**e**) fracture surface of EP/Amino-BCP/SiO_2_ at 150 °C, and (**f**) fracture surface of EP/Carboxyl-BCP/SiO_2_ at 150 °C.

Additionally, the fracture surface of the silica-filled epoxy system was investigated (Figure 10). At room temperature, the fracture surface differences between EP/SiO_2_ and EP/amino-BCP/SiO_2_ were not significant, and there was a large amount of residual matrix on the surface of the SiO_2_. However, on the fracture surface of EP/carboxyl-BCP/SiO_2_, most of the SiO_2_ surface was relatively smooth, and the dispersed phase size of the carboxyl system was significantly smaller than that prior to the introduction of SiO_2_. The fracture surface differences between the three groups of samples tested at high temperatures were generally small. However, by comparing the surfaces of the samples at different test temperatures, it was found that the phenomena of debonding and void growth were very common at room temperature. At high temperatures, most of the silica deboned directly, and voids caused by filler extrusion and matrix deformation could hardly be observed. Combined with the storage modulus in thermomechanical analysis, it can be seen that when the temperature of the samples reaches 150 °C, the chain segment movement is easily opened, causing the storage modulus of the epoxy resin to significantly reduce to less than 10 MPa. The matrix undergoes highly elastic deformation under shear conditions, forming the shear morphology in the cross-section. Due to the low modulus, even if the deformation of the matrix on the cross-section is significantly greater than that of the cross-section at room temperature, the energy that can be stored based on the deformation is significantly reduced, resulting in the fracture toughness of various systems at high temperatures being generally small and basically consistent.

**Figure 11:**
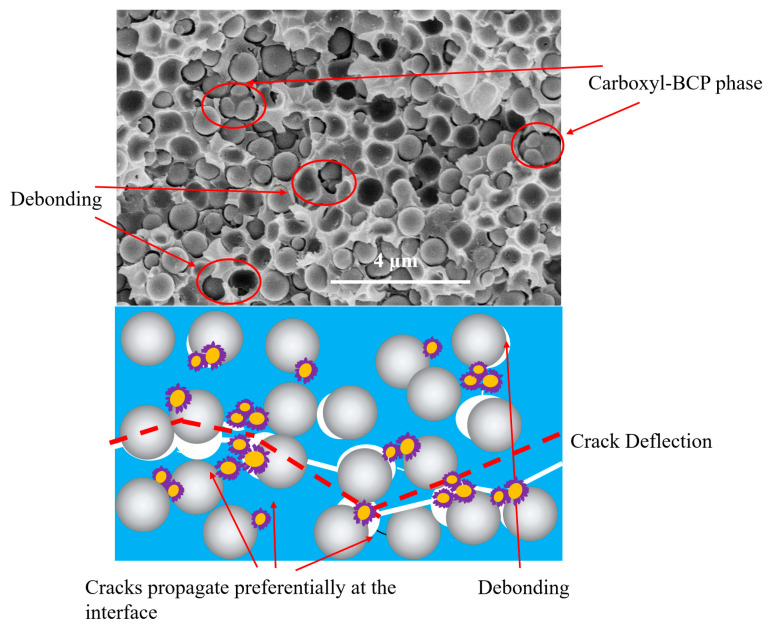
Proposed mechanism for explaining the increase in fracture toughness of EP/Carboxyl-BCP/SiO_2_.

After further analysis of the fracture surface of EP/carboxyl-BCP/SiO_2_ tested at room temperature, the cause of the decrease in the fracture toughness of the system after the introduction of 60 wt.% SiO_2_ was found. As shown in Figure 11, when a large amount of SiO_2_ was introduced, the island phase became smaller and was distributed around the SiO_2_, disrupting the original resin network system. This caused cracks to propagate preferentially along the deboned void edge rather than within the matrix. 

### 3.5. Strain Field at the Crack Tip

**Figure 12:**
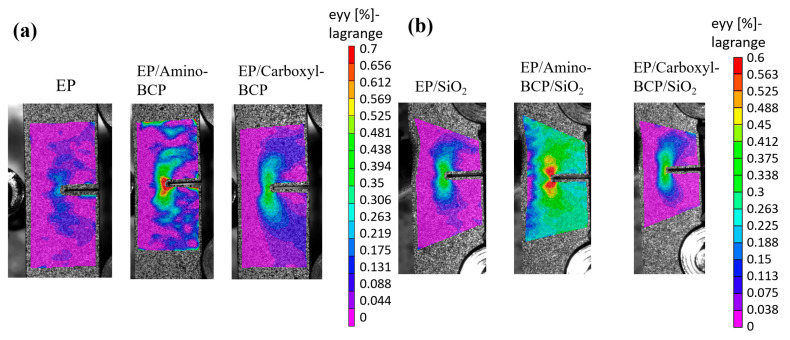
Results of crack-tip strain of (**a**) epoxy and epoxy/BCP system at maximum load, (**b**) epoxy and epoxy/BCP with 60 wt.% SiO_2_ system at maximum load.

Using DIC technology, in situ observations of the crack propagation process at room temperature were performed. The strain distribution of the spline was obtained when it reached its maximum load, compared with the initial state at zero load. As shown in Figure 12a, the results of the crack-tip strain of the epoxy and epoxy/BCP systems were consistent with the previous discussion. The distributions of the strain field and the value at the crack tip of the EP/amino-BCP were higher, indicating that the system was capable of absorbing higher strain energy. This is closely related to the effective stress transmission of the block copolymers as flexible segments in the matrix.

The strain distribution in the silica-filled epoxy system was also observed (Figure 12b), and the results were similar. Owing to the introduction of SiO_2_ and BCP, the network structure of the epoxy resin changed, and the molecular chains of the different systems underwent changes to their maximum strain. Generally, the wider the strain distribution, the greater the strain, the more fracture energy absorbed, and the better the fracture toughness.

## 4. Conclusions

This work proved that amino-terminated BCPs had the most obvious effect on the fracture toughness of an epoxy resin-amine curing system at room temperature. Due to the effective stress transmission of block copolymers as flexible segments in the matrix, compared with the control group, the fracture toughness at room temperature increased by 68.9% without adding SiO_2_; after 60 wt.% SiO_2_ was introduced, the fracture toughness at room temperature increased by 31.2%. Additionally, the fracture toughness of the epoxy resin system at high temperatures was not affected by the terminal groups of block copolymers. In terms of thermal mechanical properties, the storage modulus and Tg of EP/Amino-BCP were basically not affected, but there was a slight amplification effect on CTE1. Compared with the control group, there was an increase of 4.8 ppm/K in CTE1 without the introduction of silica; after 60 wt.% SiO_2_ was introduced, there was an increase of 6.1 ppm/K in CTE1. In addition, based on the characterization results of SEM and DIC, it was found that the SiO_2_/Epoxy de-bonding, void growth, and the stress transmission of the block copolymer are the reasons for toughening. The discussion of results in this work provide an in-depth understanding of the effect of the different toughening agents on material properties and, therefore, provide guidance for the design of epoxy-resin-based composites in underfill applications.

## Figures and Tables

**Table 1:** Sample label comparison table.

Sample	Abbreviation Label
Neat epoxy	EP
Epoxy/Amino-terminated BCP	EP/Amino-BCP
Epoxy/Carboxyl-terminated BCP	EP/Carboxyl-BCP
Epoxy/60 wt.% SiO_2_	EP/SiO_2_
Epoxy/Amino-terminated BCP/60 wt.% SiO_2_	EP/Amino-BCP/SiO_2_
Epoxy/Carboxyl-terminated BCP/60 wt.% SiO_2_	EP/Carboxyl-BCP/SiO_2_

**Table 2:** Thermal expansion coefficient of epoxy/BCPs with 0% and 60 wt.% silica.

	CTE 1/(ppm/K)	CTE 2/(ppm/K)
EP	65.8	189.8
EP/Amino-BCP	70.6	178.7
EP/Carboxyl-BCP	69.6	182.7
EP/SiO_2_	29.6	103.8
EP/Amino-BCP/SiO_2_	35.7	103.3
EP/Carboxyl-BCP/SiO_2_	32.5	102.8

## Data Availability

The data presented in this study are available from the corresponding author upon request.

## References

[1] Zucchi I.A., Galante M.J., Williams R.J.J. (2005). Comparison of morphologies and mechanical properties of crosslinked epoxies modified by polystyrene and poly(methyl methacrylate)) or by the corresponding block copolymer polystyrene-b-poly(methyl methacrylate). Polymer.

[2] Wetzel B., Rosso P., Haupert F., Friedrich K. (2006). Epoxy nanocomposites-fracture and toughening mechanisms. Eng. Fract. Mech..

[3] Li J., Liu S., Liang H., Hao L., Wang Y., Du B. (2023). Study on non-uniformity and dynamic fracture characteristics of GIL tri-post insulators considering Al2O3 sedimentation. High Volt..

[4] Pecora M., Pannier Y., Lafarie-Frenot M., Gigliotti M., Guigon C. (2016). Effect of thermo-oxidation on the failure properties of an epoxy resin. Polym. Test.

[5] Dong K., Zhang J., Cao M., Wang M., Gu B., Sun B. (2016). A mesoscale study of thermal expansion behaviors of epoxy resin and carbon fiber/epoxy unidirectional composites based on periodic temperature and displacement boundary conditions. Polym. Test.

[6] Xiong X., Zhou L., Ren R., Ma X., Chen P. (2018). Thermal, mechanical properties and shape memory performance of a novel phthalide-containing epoxy resins. Polymer.

[7] Jung H., Park Y., Nah C., Lee J., Kim K., Lee C.S. (2021). Evaluation of the Mechanical Properties of Polyether Sul-fone-Toughened Epoxy Resin for Carbon Fiber Composites. Fiber Polym..

[8] Mathew V.S., Jyotishkumar P., George S.C., Gopalakrishnan P., Delbreilh L., Saiter J.M., Saikia P.J., Thomas S. (2012). High Performance HTLNR/Epoxy Blend-Phase Morphology and Thermomechanical Properties. J. Appl. Polym. Sci..

[9] An H., Liu Z., Tian Q., Li J., Zhou C., Liu X., Zhu W. (2019). Thermal behaviors of nanoparticle reinforced epoxy resins for mi-croelectronics packaging. Microelectron. Reliab..

[10] Hsieh T.H., Kinloch A.J., Masania K., Taylor A.C., Sprenger S. (2010). The mechanisms and mechanics of the toughening of epoxy polymers modified with silica nanoparticles. Polymer.

[11] Lee M., Paria S., Mondal S., Lee G., Shin B., Kim S., Park S., Nah C. (2022). Amphiphilic block co-polymer and silica reinforced epoxy composite with excellent toughness and delamination resistance for durable electronic packaging application. Polymer.

[12] Kinloch A.J., Shaw S.J., Tod D.A., Hunston D.L. (1983). Deformation and fracture behaviour of a rubber-toughened epoxy: 1. Mi-crostructure and fracture studies. Polymer.

[13] Kishi H., Shi Y.B., Huang J., Yee A.F. (1998). Ductility and toughenability study of epoxy resins under multiaxial stress states. J. Mater. Sci..

[14] Wang M., Fan X., Thitsartarn W., He C. (2015). Rheological and mechanical properties of epoxy/clay nanocomposites with en-hanced tensile and fracture toughnesses. Polymer.

[15] Cho D., Hwang J.H. (2013). Elastomeric Coating of Exfoliated Graphite Nanoplatelets with Amine-Terminated Poly(butadiene-co-acrylonitrile): Characterization and Its Epoxy Toughening Effect. Adv. Polym. Technol..

[16] Mijovic J., Shen M., Sy J.W., Mondragon I. (2000). Dynamics and Morphology in Nanostructured Thermoset Network/Block Co-polymer Blends during Network Formation. Macromolecules.

[17] Grishchuk S., Sorochynska L., Vorster O.C., Karger-Kocsis J. (2013). Structure, thermal, and mechanical properties of DDM-hardened epoxy/benzoxazine hybrids: Effects of epoxy resin functionality and ETBN toughening. J. Appl. Polym. Sci..

[18] Konnola R., Parameswaranpillai J., Joseph K. (2016). Mechanical, thermal, and viscoelastic response of novel in situ CTBN/POSS/epoxy hybrid composite system. Polym. Compos..

[19] Song X., Xu S. (2016). Curing kinetics of pre-crosslinked carboxyl-terminated butadiene acrylonitrile (CTBN) modified epoxy blends. J. Therm. Anal. Calorim..

[20] Vijayan P.P., Puglia D., Vijayan P.P., Kenny J.M., Thomas S. (2017). The role of clay modifier on cure characteristics and proper-ties of epoxy/clay/carboxyl-terminated poly(butadiene-co-acrylonitrile) (CTBN) hybrid. Mater. Technol..

[21] Bucknall C.B., Gilbert A.H. (1989). Toughening tetrafunctional epoxy resins using polyetherimide. Polymer.

[22] Raghava R.S. (1987). Role of matrix-particle interface adhesion on fracture toughness of dual phase epoxy-polyethersulfone blend. J. Polym. Sci. Part B Polym. Phys..

[23] Keller A., Chong H.M., Taylor A.C., Dransfeld C., Masania K. (2017). Core-shell rubber nanoparticle reinforcement and pro-cessing of high toughness fast-curing epoxy composites. Compos. Sci. Technol..

[24] Jingqiang S., Yafeng Z., Jindong Q., Jianzheng K. (2004). Core-shell particles with an acrylate polyurethane core as tougheners for epoxy resins. J. Mater. Sci..

[25] Giannakopoulos G., Masania K., Taylor A.C. (2011). Toughening of epoxy using core-shell particles. J. Mater. Sci..

[26] Staudinger U., Satapathy B.K., Weidisch R. (2008). Influence of block composition on crack toughness behaviour of sty-rene-b-(styrene-random-butadiene)-b-styrene triblock copolymers. Eur. Polym. J..

[27] Cong H., Li L., Zheng S. (2014). Formation of nanostructures in thermosets containing block copolymers: From self-assembly to reaction-induced microphase separation mechanism. Polymer.

[28] Rio T.G., Salazar A., Pearson R.A., Rodriguez J. (2016). Fracture behaviour of epoxy nanocomposites modified with triblock co-polymers and carbon nanotubes. Compos. Part B-Eng..

[29] Li M., Heng Z., Chen Y., Zou H., Liang M. (2018). High Toughness Induced by Wormlike-Nanostructure in Epoxy Thermoset Containing Amphiphilic PDMS-PCL Block Copolymers. Ind. Eng. Chem. Res..

[30] Ding H., Zhao B., Mei H., Li L., Zheng S. (2019). Toughening of epoxy thermosets with polysty-rene-block-polybutadiene-block-polystyrene triblock copolymer via formation of nanostructures. Polym. Eng. Sci..

[31] Zhou Q., Liu Q., Yu Y., Zhuang Y., Lv Y., Xiao H., Song N., Ni L. (2020). Morphological evolution and mechanical properties of an “anchor chain” nanodomain structure of a reactive amphiphilic triblock copolymer in epoxy resin. Polym. Chem..

[32] Silva B.L., Schuster M.B., Bello R.H., Becker D., Coelho L.A.F. (2020). The role of carbon nanoparticles in epoxy-based nano-composites modified with (poly[polypropylene oxide]-block-poly[ethylene oxide]-block-poly[propylene oxide]) triblock co-polymers on phase morphology and mechanical properties. Polym. Compos..

[33] Tao L., Sun Z., Min W., Ou H., Qi L., Yu M. (2020). Improving the toughness of thermosetting epoxy resins via blending triblock copolymers. RSC Adv..

[34] Jheng L.C., Wang I.H., Hsieh T.H., Fan C.T., Hsiao C.H., Wu C.P., Leu M.T., Chang T.Y. (2021). Toughening of epoxy thermo-sets with nano-sized or micron-sized domains of poly (ethylene oxide)-b-poly(butadiene-co-acrylonitrile)-b-poly(ethylene oxide) triblock copolymers synthesized using room temperature ester coupling reaction. J. Appl. Polym. Sci..

[35] Silva B.L., Schuster M.B., Becker D., Coelho L.A.F. (2022). The Influence of the Molecular Architecture of the PEG: PPG Triblock Copolymer on the Properties of Epoxy Nanocomposites. Mater. Res..

[36] Li L., Peng W., Liu L., Zheng S. (2022). Toughening of epoxy by nanostructures with ABA triblock copolymers: An influence of organosilicon modification of block copolymer. Polym. Eng. Sci..

[37] Cano L., Gutierrez J., Tercjak A. (2015). Enhancement of the mechanical properties at the macro and nano scale of thermosetting systems modified with a polystyrene-block-polymethylmethacrylate block copolymer. RSC Adv..

[38] Paz E., Abenojar J., Ballesteros Y., Forriol F., Dunne N., Del Real J.C. (2016). Mechanical and thermal behaviour of an acrylic bone cement modified with a triblock copolymer. J. Mater. Sci. Mater. Med..

[39] Ignaczak W., Wiśniewska K., Janik J., Fray M.E. (2015). Mechanical and thermal properties of PP/PBT blends compatibilized with triblock thermoplastic elastomer. Pol. J. Chem. Technol..

[40] Kishi H., Kunimitsu Y., Nakashima Y., Imade J., Oshita S., Morishita Y., Asada M. (2017). Relationship between the mechanical properties of epoxy/PMMA-b-PnBA-b-PMMA block copolymer blends and their three-dimensional nanostructures. Express Polym. Lett..

[41] Mao H.I., Hsu T.S., Chen C.W., Huang K.W., Rwei S.P. (2022). Synthesis and characteristics of poly(ethylene terephthalate) with EO-PO-EO triblock copolymers: A thermal and mechanical property study. J. Appl. Polym. Sci..

[42] Asada M., Oshita S., Morishita Y., Nakashima Y., Kunimitsu Y., Kishi H. (2016). Effect of miscible PMMA chain length on disor-dered morphologies in epoxy/PMMA-b-PnBA-b-PMMA blends by in situ simultaneous SAXS/DSC. Polymer.

[43] Klingler A., Wetzel B. (2017). Fatigue crack propagation in triblock copolymer toughened epoxy nanocomposites. Polym. Eng. Sci..

[44] Kishi H., Kunimitsu Y., Imade J., Oshita S., Morishita Y., Asada M. (2011). Nano-phase structures and mechanical properties of epoxy/acryl triblock copolymer alloys. Polymer.

[45] George S.M., Puglia D., Kenny J.M., Parameswaranpillai J., Thomas S. (2014). Reaction-Induced Phase Separation and Thermo-mechanical Properties in Epoxidized Styrene-block-butadiene-block-styrene Triblock Copolymer Modified Epoxy/DDM System. Ind. Eng. Chem. Res..

[46] George S.M., Puglia D., Kenny J.M., Causin V., Parameswaranpillai J., Thomas S. (2013). Morphological and Mechanical Char-acterization of Nanostructured Thermosets from Epoxy and Styrene-block-Butadiene-block-Styrene Triblock Copolymer. Ind. Eng. Chem. Res..

[47] Chen J., Taylor A.C. (2012). Epoxy modified with triblock copolymers: Morphology, mechanical properties and fracture mechanisms. J. Mater. Sci..

[48] (2011). Standard Test Method for Measurement of Fracture Toughness.

[49] Carroll J.D., Abuzaid W., Lambros J., Sehitoglu H. (2013). High resolution digital image correlation measurements of strain accu-mulation in fatigue crack growth. Int. J. Fatigue.

[50] Yang Y., Souare P.M., Sylvestre J. (2020). Using Confocal Microscopy and Digital Image Correlation to Measure Local Strains Around a Chip Corner and a Crack Front. IEEE Trans. Device Mater. Reliab..

[51] Wang D., Zhu M., Xuan F. (2017). Crack tip strain evolution and crack closure during overload of a growing fatigue crack. Frat. Integrità Strutt..

